# Amphidinol 3 preferentially binds to cholesterol in disordered domains and disrupts membrane phase separation

**DOI:** 10.1016/j.bbrep.2021.100941

**Published:** 2021-02-10

**Authors:** Manami Hieda, Akira Sorada, Masanao Kinoshita, Nobuaki Matsumori

**Affiliations:** Department of Chemistry, Graduate School of Science, Kyushu University, 744 Motooka, Nishi-ku, Fukuoka, 819-0395, Japan

**Keywords:** Amphidinol 3, Cholesterol, Membrane, Fluorescence microscope, Lipid rafts, Phase separation

## Abstract

Amphidinol 3 (AM3), a polyhydroxy-polyene metabolite from the dinoflagellate *Amphidinium klebsii*, possesses potent antifungal activity. AM3 is known to interact directly with membrane sterols and permeabilize membranes by forming pores. Because AM3 binds to sterols such as cholesterol and ergosterol, it can be assumed that AM3 has some impact on lipid rafts, which are membrane domains rich in sphingolipids and cholesterol. Hence, we first examined the effect of AM3 on phase-separated liposomes, in which raft-like ordered and non-raft-like disordered domains are segregated. Consequently, AM3 disrupted the phase separation at 22 μM, as in the case of methyl-β-cyclodextrin, a well-known raft-disrupter that extracts sterol from membranes. The surface plasmon resonance measurements and dye leakage assays show that AM3 preferentially recognizes cholesterol in the disordered membrane, which may reflect a weaker lipid-cholesterol interaction in disordered membrane than in ordered membrane. Finally, to gain insight into the AM3-induced coalescence of membrane phases, we measured membrane fluidity using fluorescence correlation spectroscopy, demonstrating that AM3 significantly increases the order of disordered phase. Together, AM3 preferentially binds to the disordered phase rather than the ordered phase, and enhances the order of the disordered phase, consequently blending the separated phases.

## Highlights

• Amphidinol 3 (AM3) is an antifungal agent isolated from a marine dinoflagellate.
• AM3 merged the coexistent ordered and disordered membrane domains.
• AM3 preferentially interacted with cholesterol in disordered domain.
• Pore formation of AM3 was also more prominent in disordered membrane domain.
• AM3 reduces the fluidity of disordered domain, blending the separated phases.

## Graphical abstract

**Figure:**
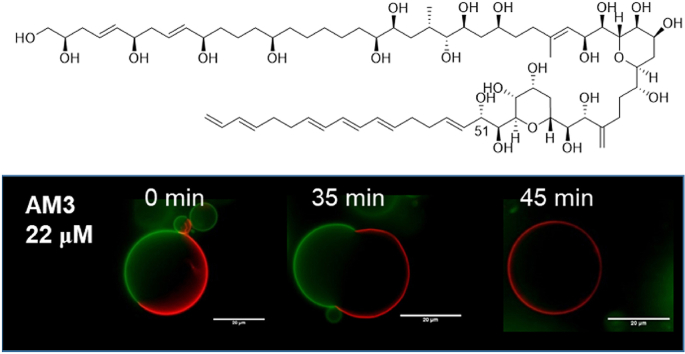

## 1 Introduction

Dinoflagellates belonging to the genus *Amphidinium* are a rich source of polyketide metabolites with unique and fascinating structures and bioactivities. In 1991, amphidinol 1, the very first member of the polyhydroxy-polyene metabolite family, was isolated from *Amphidinium klebsii* [1]. Since then, more than 20 closely related homologs, collectively termed amphidinols, have been reported [[2], [3], [4], [5], [6], [7], [8], [9], [10], [11], [12]]. In addition, numerous amphidinol analogs such as luteophanols [13] and karlotoxins [14] were isolated both from *Amphidinium* and other dinoflagellate species. These natural compounds possess polyhydroxy and polyene chains, which are separated by two tetrahydropyran rings. This structural feature confers them amphiphilic nature. The middle region of the molecule containing the tetrahydropyran rings is conserved among the amphidinols, while the structural variation mainly occurs on both the polyhydroxy and polyene chains [8,9,13].

**Fig. 1:**
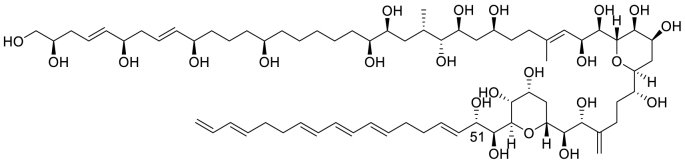
Chemical structure of AM3. The stereochemistry was unambiguously elucidated by Oishi's synthetic studies [[19], [20], [21]].

Amphidinols exhibit antifungal and hemolytic actions, which arise from their interaction with lipid bilayers, ultimately enhancing membrane permeability [3,4]. Amphidinol 3 (AM3, Fig. 1) [4,5,15] has the most potent antifungal and hemolytic activities among amphidinols. It was reported that AM3 exhibits potent pore-forming activity on liposomes [4,15], which absolutely depends on the presence of membrane sterol; *i.e.*, its absence renders AM3 inactive [15,16]. Our previous study using surface plasmon resonance (SPR) and solid-state ^2^H NMR demonstrated that AM3 directly interacts with sterols in membranes [17]. In addition, more recent channel recording experiments further suggest that AM3 forms both barrel-stave and toroidal pores depending on the AM3 concentration; a higher concentration of AM3 forms jumbo toroidal pores, while a lower concentration of AM3 forms a barrel-stave channel that shows a single channel property [18].

On the other hand, there are many sterol-binding bioactive natural products, such as polyenemacrolide antibiotics and saponins. Some of them are suggested to elicit their biological activities in association with lipid rafts, which are membrane micro-domains consisting of sphingolipids, sterols, and/or proteins [22]. For example, saponins are assumed to interact with cholesterol-enriched lipid rafts, either disrupting them or causing their miscibility [23]. Similarly, filipin III, a representative polyene macrolide, interacts with membrane sterol, causing disruption of lipid rafts or enhancement of membrane permeability [24,25]. Because it is believed that lipid rafts modulate the activity and location of various membrane receptors and consequently influence signal transduction pathways [22,[25], [26], [27]], the action of sterol-binding natural products on lipid rafts may provoke significant biological responses. Hence as the first step of this study, we examined the effect of AM3, a strong sterol-binder, on lipid membranes that have a mixture of liquid-ordered (Lo) and liquid-disordered (Ld) phases as a model for lipid rafts in cellular membranes. Subsequent experiments reveal that AM3 disrupts the membrane phase separation by preferentially binding to the disordered phase.

## 2 Materials and methods

### 2.1 Materials

Porcine brain sphingomyelin (SM) and 1,2-dioleoyl-sn-glycero-3-phosphocholine (DOPC) were purchased from Avanti Polar Lipids (Alabaster, AL). Cholesterol (Chol) was purchased from Sigma Aldrich (St. Louis, MO). Texas Red-DPPE (TXred-DPPE) was purchased from Invitrogen (Eugene, OR). Fluorescent-labeled lipids, 488neg-SM, 488neg-DOPC, 594neg-SM, and 594neg-DOPC, were synthesized following our previous report [28].

### 2.2 Isolation of AM3

The culture of the dinoflagellate *Amphidinium klebsii* and the isolation of AM3 were performed as reported previously [15].

### 2.3 Giant unilamellar vesicle (GUV) preparation and fluorescence microscopy observation

GUVs were obtained by electroformation as described by Angelova and Dimitrov [29]. In brief, SM, DOPC, and Chol (1:1:1 in molar ratio) were dissolved in CHCl_3_–MeOH (4:1v/v) to a final phospholipid concentration of 1 mg mL^−1^, to which TXred-DPPE and 488neg-SM (0.2 mol% of total lipids) were added. Aliquots (10 μL) were subsequently deposited on parallel aligned electrodes (Pt wires, 100 μm in diameter) attached to a glass slide (24 mm × 60 mm, 0.12–0.17 mm thickness), after which the solvent was evaporated under vacuum for more than 12 h. Milli-Q water (400 μL, Simplicity UV) was then added to completely immerse the electrodes, which were then sealed with another glass slide using a rubber spacer with a small fill port for AM3 injection. This chamber was maintained at 70 °C on a temperature-controlled aluminum block (Sahara 310, Rocker Scientific Co., Ltd., Taipei, Taiwan), and an alternating current (10 V, 10 Hz) was applied (20 MHz function/arbitrary waveform function generator, Agilent, Santa Clara CA) for 60 min to form GUVs. The GUVs were then cooled to 25 °C. Fluorescence microscopy observation was carried out using the BZ-X700 (Keyence, Osaka, Japan) with an air objective lens (Plan Apoλ, 60 × , N.A. 0.95, Nikon, Tokyo, Japan). The excitation (470 nm) and detection (525 nm) wavelengths were selected by dichroic mirrors, OP-87763 (Keyence, Osaka, Japan). After the GUVs without AM3 were observed, AM3 aqueous solution was added to the GUVs at the final concentrations of 11, 22, and 44 μM, and the observation was continued for 50 min.

### 2.4 SPR analysis

SPR measurements were performed at 25 °C using Biacore T100 system (GE Healthcare, Chicago, IL, USA). LUVs (DOPC, DOPC/Chol 9:1 and 7:3, SM, SM/Chol 9:1 and 7:3) were prepared using HBS-N buffer [10 mM HEPES buffer (pH 7.4), 150 mM NaCl] and immobilized on the CM5 sensor chip surface, as previously described [17]. To compensate the difference in the immobilization amount of liposomes, the immobilization was normalized to 12000 RU. AM3 solutions in HBS-N buffer (30, 40, and 50 μM) were then injected at a flow rate of 10 μL min^−1^, and the association of AM3 was monitored for 300 s. Then HBS-N running buffer was injected at the same flow rate, and the dissociation of AM3 from the surface was monitored for 300 s. The sensor chip surface was regenerated after each analysis using our previously described protocol [17].

### 2.5 Calcein leakage experiments

The extent of calcein leakage from liposomes was assessed as reported previously [15]. Large unilamellar vesicles (LUVs) were prepared as follows: DOPC (10 mg) or SM (10 mg), with or without sterol (10 or 30 mol%), was dissolved in CHCl_3_ in a round-bottom flask. The solvent was removed by nitrogen gas flow and further dried *in vacuo* for 12 h. The lipid film obtained was rehydrated with 1 mL of 60 mM calcein in HBS-N [10 mM HEPES buffer (pH 7.4), 150 mM NaCl] and subjected to two cycles of vortexing (1 min) and warming (65 °C) followed by five cycles of freezing (−20 °C) and thawing (65 °C) to obtain multilamellar vesicles (MLVs). Then, the suspension was passed through a polycarbonate membrane filter (pore size, 200 nm) 19 times using a Mini-Extruder (Avanti Polar Lipids, Inc.) to prepare LUVs of homogenous size. Excess calcein was removed by passing the suspension through a Sephadex G-75 column (Sigma-Aldrich) with HBS-N buffer. The lipid concentration in the LUV fraction was quantified using phospholipid C-Test Wako (Wako Pure Chemical Industries, Ltd., Osaka, Japan). Resulting stock solution was stored at 4 °C under nitrogen gas. Measurement of calcein leakage was performed on a JASCO FP 8300 spectrofluorometer (JASCO Corp., Tokyo, Japan) with an excitation wavelength of 490 nm and an emission wavelength of 517 nm. To monitor calcein leakage, the LUV suspension was diluted to 980 μL with the HBS-N buffer, and a 20-μL aliquot of AM3 in HBS-N buffer was then added to give the final AM3 concentration of 0, 1, 5, or 10 μM. Subsequently, 20 μL of 10% Triton X-100 (v/v) (Nacalai Tesque, Kyoto, Japan) was added to obtain the condition of 100% leakage. All measurements were performed at room temperature with a final lipid concentration of 27 μM.

### 2.6 Fluorescence correlation spectroscopy (FCS)

The GUVs for FCS measurements were prepared in the manner as described above. For the observation of Fig. 5, DOPC/Chol (9:1) and SM/Chol (9:1) GUVs containing 0.002 mol% 594neg-DOPC and 594neg-SM, respectively, were prepared. The GUVs used for Fig. 6 observation were composed of SM/DOPC/Chol (1:1:1) and contained 0.002 mol% 594neg-SM (for the Lo phase observation) and 594neg-DOPC (for the Ld phase observation). FCS measurements started 15 min after AM3 dissolved in HBS-N buffer was extraneously added to the GUVs. The measurements were performed at 25 °C with a confocal microscope (FV1000D, IX81; Olympus, Tokyo, Japan), using an oil-immersion apochromat objective lens (Olympus PLAPON60XO, 60 × , NA 1.4). Diffusion coefficients were obtained following the protocol published previously [30,31]. The diffusion coefficient, *D*, was obtained by fitting the autocorrelation function of the time-dependent changes of the signal intensities of fluorescent probe molecules in diffraction-limited spots, *G*(*τ*), with the following equation for two-dimensional simple Brownian diffusion:

$$G\left(\tau \right)=\frac{1}{N}\left\{\frac{1}{1+\left(\frac{4D\tau }{w_{0}^{2}}\right)}\right\}\text{,}$$

where *N* is the average number of fluorescent particles in the detection area. The beam waist (radius) in the focal plane *w*_0_ (= 0.14 and 0.16 μm for excitation wavelengths of 488 and 560 nm, respectively) was calibrated with Rhodamine 6G, for which the diffusion coefficient is known. *D* is the diffusion coefficient, and *τ* is the delay time.

**Fig. 2:**
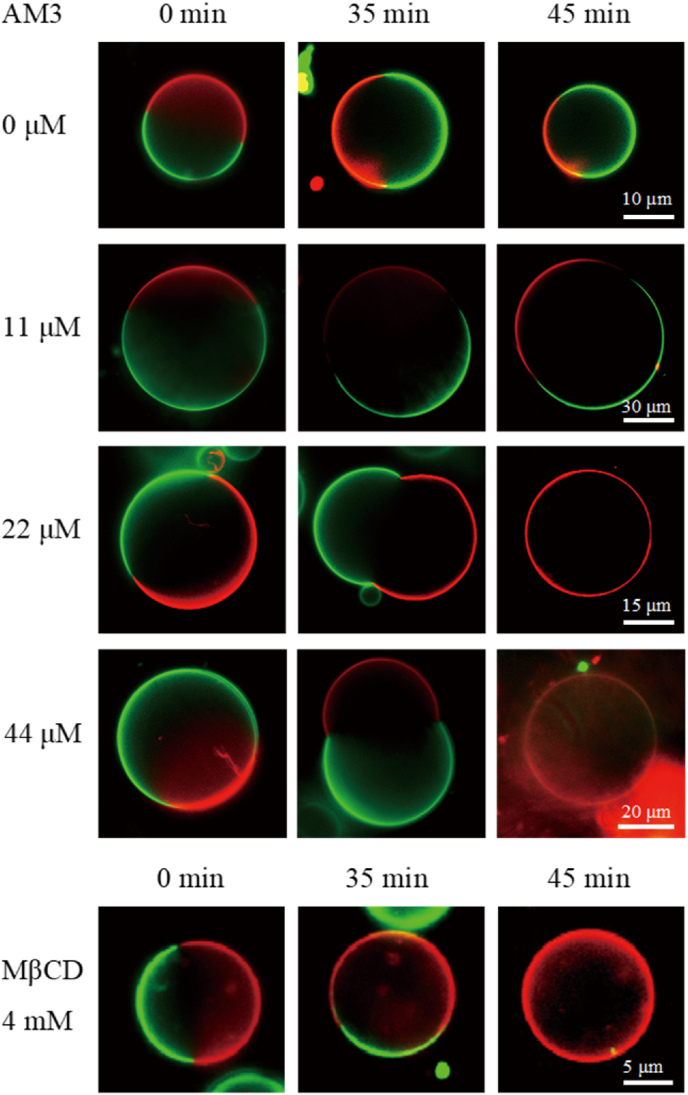
Fluorescence microscopy observation of phase-separated GUVs in the presence of AM3 and MβCD. GUVs were composed of SM/DOPC/Chol (1:1:1). The disordered and ordered phases were labeled with 0.2 mol% TexRed-DPPE (Ld marker, red) and 488neg-SM (Lo marker, green), respectively. The green fluorescence of 488neg-SM was hardly observed after the two phases became miscible at 22 and 44 μM AM3 or 4 mM MβCD, because the green fluorescence was quenched by the FRET with TexRed-DPPE. (For interpretation of the references to colour in this figure legend, the reader is referred to the Web version of this article.)

**Fig. 3:**
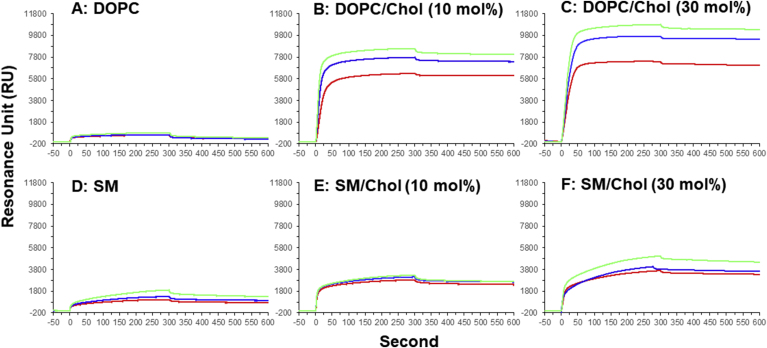
SPR sensorgrams for the binding of AM3 to the liposomes immobilized on a dodecylamine-modified CM5 sensor chip: DOPC liposomes in the absence or presence of 10 and 30 mol % Chol (A, B, and C), and SM liposomes in the absence or presence of 10 and 30 mol % Chol (D, E, and F). The concentrations of AM3 are 30 (red), 40 (blue), and 50 (green) μM. The association of AM3 was monitored from 0 to 300 s, and its dissociation from the surface was recorded from 300 to 600 s. (For interpretation of the references to colour in this figure legend, the reader is referred to the Web version of this article.)

**Fig. 4:**
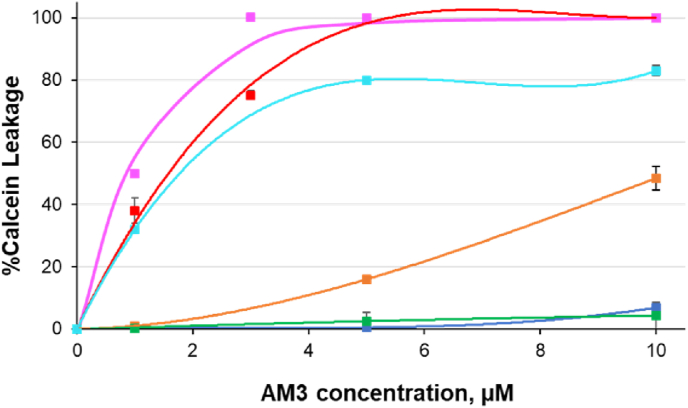
AM3-induced calcein leakage from DOPC/Chol (7:3; pink, 9:1; red), SM/Chol (7:3; pale blue, 9:1; orange), DOPC (green), and SM (blue) liposomes. In all cases, the final lipid concentration was 27 μM. (For interpretation of the references to colour in this figure legend, the reader is referred to the Web version of this article.)

**Fig. 5:**
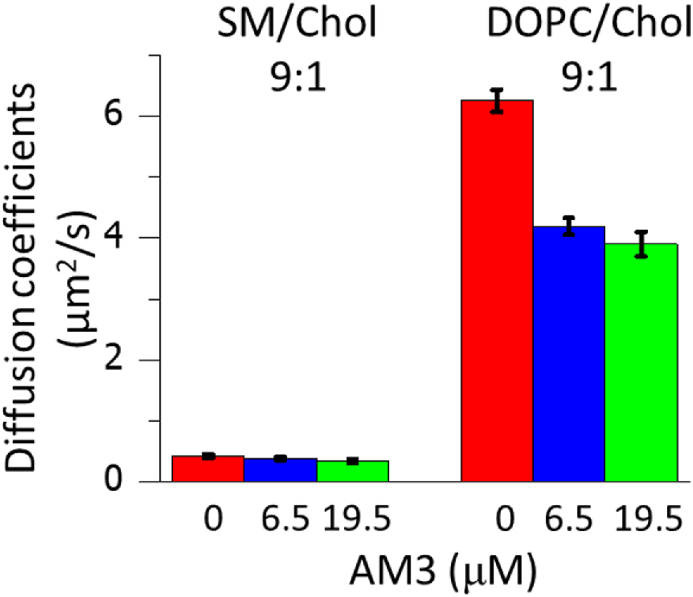
Diffusion coefficients of 594neg-SM in SM/Chol (9:1) GUV, and 594neg-DOPC in DOPC/Chol (9:1) GUV, determined by FCS. Error bars indicate standard errors (*n* = 23–28 GUVs). 6.5 μM of AM3 corresponds to twice the amount of Chol in GUVs.

**Fig. 6:**
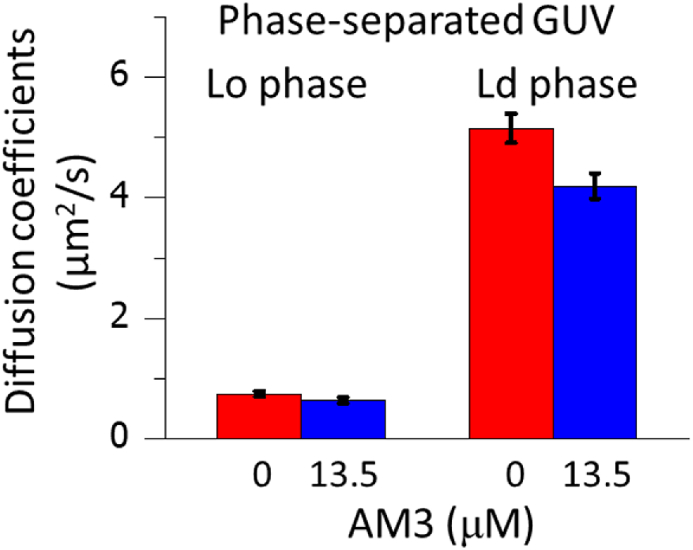
Diffusion coefficients of 594neg-SM (Lo marker) and 594neg-DOPC (Ld marker) in Lo-Ld phase-separated GUVs composed of SM/DOPC/Chol (1:1:1), determined by FCS. Error bars indicate standard errors (*n* = 20–39 GUVs). 13.5 μM of AM3 is equimolar to Chol in the GUVs.

## 3 Results

### 3.1 Effect of AM3 on phase-separated membranes

To examine the effect of AM3 on Lo-Ld phase-separated membranes which mimic lipid rafts, we prepared giant unilamelar vesicles (GUVs) composed of SM, DOPC, and Chol (Fig. 2). In GUVs, the Lo and Ld phases are visualized with recently-developed 488neg-SM (green fluorescence) [28] and commercial TexRed-DPPE (red fluorescence), respectively. The addition of 11 μM of AM3 (Chol:AM3 molar ratio is 1:1) did not coalesce the separated phases, although some constriction was observed around phase boundary. Meanwhile, at 22 and 44 μM of AM3, the separated phases became miscible after 45 min. The deformation of GUVs at 35 min in the presence of 22 and 44 μM AM3 is probably the result of the increase of the outer leaflet surface area by AM3 binding. Although methyl-β-cyclodextrin (MβCD), which extracts Chol from membrane and disrupts ordered domains, also mixed the separate phases at 4 mM, the efficiency is much lower than AM3. This experiment clearly demonstrates that AM3 more effectively makes the separate phases miscible than MβCD.

### 3.2 Interaction of AM3 towards ordered and disordered membranes

To gain insight into the mechanism underlying the AM3-induced disruption of phase separation, we performed surface plasmon resonance (SPR) experiments using liposomes that mimic ordered and disordered phases. A dodecylamine-modified CM5 sensor chip was prepared to immobilize the liposomes on the sensorchip as we previously reported [16,32]. The sensorgrams (Fig. 3) show that Chol significantly enhances the binding of AM3 on the membranes, which is consistent with our previous reports [16,17]. Taking account of the fact that AM3 directly binds to Chol in lipid bilayers [17], the data clearly show that AM3 recognizes Chol existing in DOPC membrane better than the one in SM membrane.

To evaluate the SPR data more quantitatively, we extracted kinetic parameters from the sensorgrams using the two-state reaction model, which was proved to be suitable for analysis of the binding of AM3 on membranes [16]. In this model, AM3 binding is assumed to be composed of two sequential steps; the first step is binding of AM3 to the membrane surface, and the second step corresponds to the penetration of AM3 to the membrane interior to form more stable complexes probably with sterol. In Table 1, *k*_a1_ and *k*_d1_ are association and dissociation rate constants of the first step, while *k*_a2_ and *k*_d2_ are association and dissociation rate constants for the second step. Accordingly, *K*_D1_, *K*_D2_, and *K*_D_ represent the dissociation constants for the first step, the second step, and overall equilibriums, respectively. Notably, the overall dissociation constant *K*_D_ for DOPC/Chol is remarkably smaller than that for SM/Chol, in line with the above conclusion that AM3 recognizes Chol in DOPC membrane better than the one in SM membrane. The kinetic data further demonstrate that although Chol promotes both the first and second steps of AM3 binding in both the DOPC and SM membranes, the Chol's effect is more prominent in the DOPC membrane than in SM membrane, which will be discussed later.

**Table 1:** Kinetic parameters for AM3 binding to the immobilized liposomes.^a^

	DOPC	DOPC/Chol (9:1)	DOPC/Chol (7:3)	SM	SM/Chol (9:1)	SM/Chol (7:3)
*k*_a1_ ( × 10^3^/Ms)	1.49	1.90	1.39	0.770	2.42	1.72
*k*_d1_ ( × 10^−2^/s)	9.54	1.20	0.582	10.6	7.76	9.65
*k*_a2_ ( × 10^−2^/s)	1.53	1.23	1.31	1.56	1.23	1.50
*k*_d2_ ( × 10^−5^/s)	156	8.33	7.44	72.4	25.5	22.9
*K*_D1_ ( × 10^−6^/M)	64.0	6.32	4.19	138	32.1	56.1
*K*_D2_ ( × 10^−3^)	102	6.77	5.68	46.4	20.7	15.3
*K*_D_ ( × 10^−8^/M)	652	4.27	2.38	640	66.4	85.7

a The data were obtained using two state reaction model. AM3 concentration was 50 μM. Standard errors are within 5% (*n* = 3).

### 3.3 Pore formation of AM3 in ordered and disordered membranes

To examine the channel formation of AM3 in ordered and disordered membranes, we next performed calcein leakage assays using SM or DOPC membranes in the presence and absence of Chol (Fig. 4). Although AM3 expectedly displays significant pore forming activity in the presence of Chol, the activity is more prominent in DOPC/Chol system than in SM/Chol system. Because AM3 strictly requires Chol to form pores [4,[15], [16], [17]], this data show that AM3 preferentially bind to Chol existing in a less ordered DOPC membrane, which is in agreement with the aforementioned SPR experiments.

### 3.4 Membrane ordering effect of AM3 on ordered and disordered membranes

Finally, to investigate the ordering effect of AM3 on ordered and disordered membranes, we measured diffusion coefficients of lipids using fluorescence correlation spectroscopy (FCS) experiments. First, we prepared SM/Chol and DOPC/Chol membranes, which mimic the ordered and disordered phases, and labeled those membranes with 0.002 mol% 594neg-SM and 594neg-DOPC, respectively. These fluorescent lipids, which we recently developed, were shown to reproduce the diffusion properties of native lipids [28]. As a result, AM3 reduced the diffusion coefficients in the DOPC/Chol membrane more prominently than in the SM/Chol membrane (Fig. 5), suggesting that AM3 more effectively enhances the order of the disordered membrane than that of ordered membrane. It is reported that AM3 forms domain-like aggregate on the membrane [18], which may increase the membrane order.

We further examined the effect of AM3 on the order of phase-separated membranes. To do this, we prepared Lo-Ld phase-coexistent GUVs composed of SM/DOPC/Chol (1:1:1 in molar ratio) and treated the GUVs with AM3 at a concentration lower than that it disrupts the phase separation. The Lo and Ld phases in the GUVs were labeled with 594neg-SM and 594neg-DOPC, respectively, and the respective diffusion coefficient was obtained by the FCS measurements (Fig. 6). As a result, although the addition of AM3 reduces the diffusion coefficients in both the Lo and Ld phases, the reduction is more prominent in the Ld phase than in the Lo phase. This confirms that the effect of AM3 on the membrane order is larger in the Ld phase than in the Lo phase.

## 4 Discussion

It was reported that AM3's pore formation is mediated by the direct interaction between AM3 and sterol in membranes [17]. This action seems to have some resemblance to polyene macrolide antibiotics such as amphotericin B, nystatin, and filipin III. For example, filipin III is known to bind to Chol in membrane and disrupt lipid rafts [24,25] as in the case with MβCD. In addition, because filipin III is reported to accumulate in Chol-rich lipid rafts, it is sometimes used as a raft marker [33]. Hence we first expected that AM3 also preferentially interacts with raft-like ordered membrane. However, SPR and calcein leakage experiments unequivocally revealed that AM3 preferentially binds to Chol existing in a less-ordered DOPC membrane and consequently forms pores more effectively in the DOPC/Chol membrane than in the SM/Chol membrane.

Here, the difference in the Chol content in the Lo and Ld phases should be taken into account. The Chol content in the Lo phase of the phase-separated membranes is more than 30 mol%, while that in the Ld phase is less than 20 mol% [34]. This means that the concentration of Chol is roughly twice larger in Lo phase than in Ld. Therefore, although AM3 preferentially binds to Chol of the Ld phase, the larger content of Chol in the Lo domain arises the concern that a larger amount of AM3 eventually binds to the Lo domain. Although membrane binding and pore formation of AM3 are significantly enhanced in SM/Chol (7:3) membrane compared in SM/Chol (9:1) (Fig. 3, Fig. 4, Table 1), those activities are still inferior to those in DOPC/Chol (9:1). Hence, although Chol content is twice higher in the Lo domain than in the Ld, it can be said that the membrane binding and pore formation of AM3 is more prominent in the Ld domain.

To evaluate the SPR data, we adopted two-reaction model following our previous report [16]. In this model, AM3's membrane binding is assumed to be composed of two sequential steps; the first step is binding of AM3 to the membrane surface, and the second step corresponds to the penetration of AM3 to the membrane interior to form more stable complexes probably with sterol. Table 1 not only quantitatively verifies that the overall affinity of AM3 is higher to the DOPC/Chol membrane than to the SM/Chol, but also demonstrates that Chol promotes both the first and second binding steps more efficiently in DOPC membrane than in SM membrane. It is assumed that AM3 recognizes Chol at multiple sites [17,21]; the tetrahydrofurane rings of AM3 recognizes the 3-OH group of Chol and the polyene chain of AM3 binds to the hydrophobic sterol skeleton. Based on this multiple recognition between AM3 and Chol, the two-reaction model can be interpreted as follows; the first process is the contact of AM3 with the Chol's 3-OH group residing at the water/membrane interface, and the second step corresponds to the interaction between AM3 polyene chain and Chol's hydrophobic skeleton in the membrane interior. It is known that Chol's 3-OH group is positioned more deeply in SM membrane than in PC membrane due to so-called umbrella effect [35], which should retard the first process of AM3 binding in the SM/Chol membrane. In addition, the interaction between SM and Chol is believed to be much stronger than the PC-Chol interaction [36], which would further hinder the second binding process between AM3 and Chol. In other words, AM3-Chol multiple recognition both at the membrane surface and in the membrane interior proceeds more smoothly in the DOPC membrane than in the SM membrane.

Another interesting finding in this study is that AM3 disrupts the Lo-Ld phase-separation of membranes (Fig. 1). The deformation of GUVs at 35 min in the presence of 22 and 44 μM AM3 is probably the result of the increase of the outer leaflet surface area by AM3 binding. AM3 bound to the outer leaflet should give rise to asymmetric imbalance of the number of molecules between the outer and inner leaflets, which would increase the curvature in each phase, leading to the deformation of GUVs. After the Lo and Ld phases are merged, the asymmetric imbalance would be eliminated by either or both of the following two processes; one is the internalization of AM3, and the other is flip-flop of lipid molecules. Since AM3 can form toroidal-type pores [18], it is thought that lipid and AM3 molecules can move easily between the outer and inner leaflets through the pores. Similarly, the disruption of the Lo and Ld phases was observed when phase-separated membranes are treated with Chol-binding reagents such as MβCD, saponin [23], and filipin III [24]. These reagents are frequently used as raft-disputers in biological studies, although detailed mechanism underlying the raft disruption by those reagents has not been fully disclosed. We recently reported that a local anesthetic dibucaine effectively destroys the Lo-Ld phase separation, which is induced by the reduction of the order of the Lo membranes [37]. In contrast, our current FCS experiments show that AM3 significantly enhances the lipid order of disordered membranes (Fig. 5, Fig. 6) and consequently reduces the difference in membrane fluidity between Lo and Ld phases, which likely induces the raft disruption. As described above, AM3 is reported to form domain-like aggregate on the membrane [18], which would increase the membrane order. On the other hand, AM3 exerted much less ordering effect on the ordered membranes (Fig. 5, Fig. 6), probably because AM3 could not further solidify the ordered membrane, which is originally sufficiently rigid. Note that, although AM3 enhances the order of the Ld domains, the diffusion coefficient of the AM3-treated Ld phase is still much larger than that of Lo phase (Fig. 6), indicating that AM3 cannot enhance the order of the Ld phase to a comparable level to the Lo phase. Then how does AM3 disrupt the phase separation? A possible explanation is that since membrane phase separation is based on a delicate balance of lipid compositions between the Lo and Ld phases, a slight increase in membrane order of the disordered phase would cause an irreversible change in lipid distribution, triggering the disruption of phase separation.

In conclusion, we found that AM3 disrupts membrane phase separation and disclosed its mechanism of action. As mentioned in the Introduction, lipid rafts are believed to play significant functions in signal transduction, and therefore it is not far-fetched to consider that the disruption of lipid rafts induced by reagents would provoke serious biological responses. Although the biological activities of AM3 such as antifungal, hemolysis, and cytotoxicity can be mostly accounted for by its pore formation in membrane, the raft-disrupting activity of AM3 may be involved in its known and unknown activities. Since numerous sterol- and lipid-binding reagents are known to date [38], the impacts of those reagents on lipid rafts and the subsequent physiological responses would be an interesting research perspective for the future.

## CRediT authorship contribution statement

**Manami Hieda:** Investigation. **Akira Sorada:** Investigation, Visualization. **Masanao Kinoshita:** Visualization, Investigation. **Nobuaki Matsumori:** Conceptualization, Supervision, Writing.

## Declaration of competing interest

We do not have any conflict of interest.
