# Supplementary material for: Amphidinol 3 preferentially binds to cholesterol in disordered domains and disrupts membrane phase separation
Source: Biochem Biophys Rep. 2021 Feb 10;26:100941. doi: 10.1016/j.bbrep.2021.100941 (PMC7881217; doi:10.1016/j.bbrep.2021.100941)
Supplement: Supplementary file 1 — Multimedia component 1 [file mmc1.pdf]

Supporting information

Amphidinol 3 preferentially binds to cholesterol in disordered domains and disrupts membrane phase separation

*Manami Hieda, Akira Sorada, Masanao Kinoshita, and Nobuaki Matsumori,**

matsmori@chem.kyushu-univ.jp

Department of Chemistry, Graduate School of Science, Kyushu University,

744 Motooka, Nishi-ku, Fukuoka 819-0395, Japan

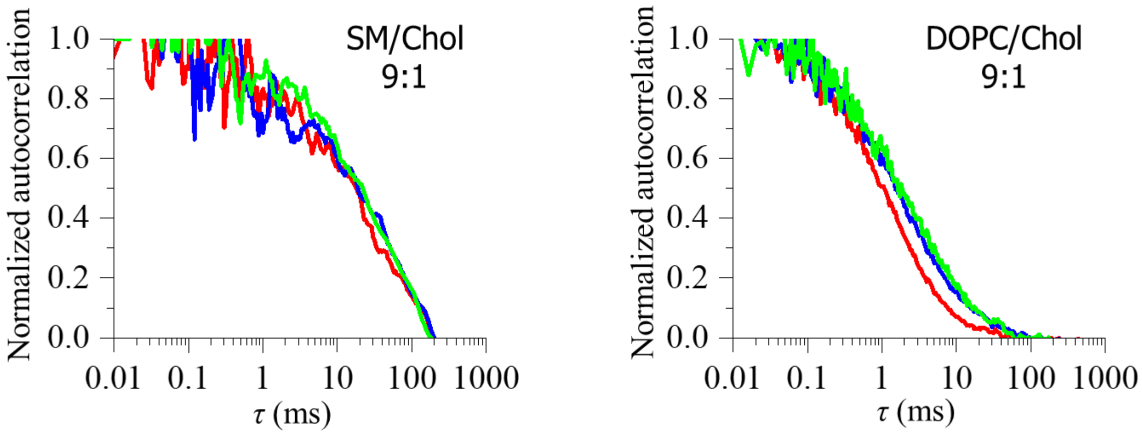

Figure S1. Representative FCS curves of 594neg-SM in SM/Chol (9:1) GUV (left panel) and 594neg-DOPC DOPC/Chol (9:1) GUVs (right panel) in the absence (red) and presence of AM3 (2.6 nmol in blue, and 7.8 nmol in green).

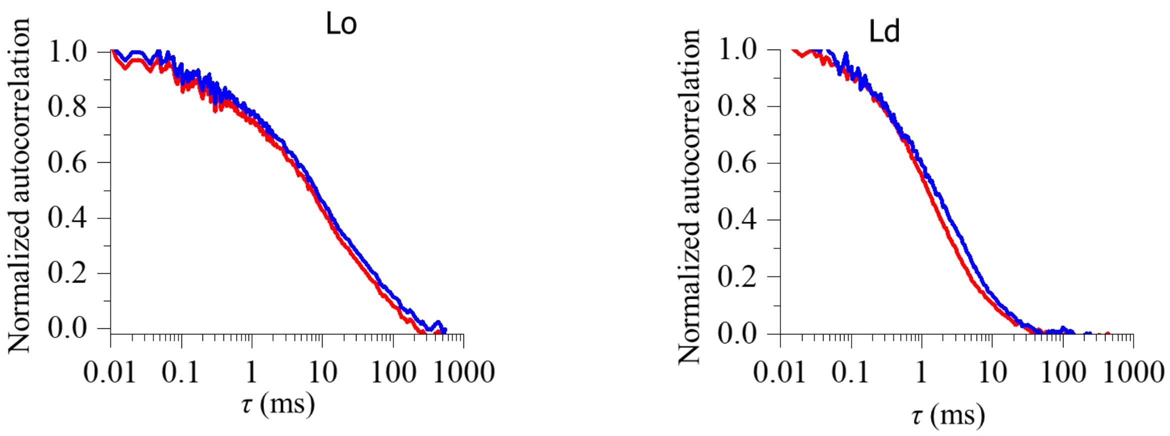

Figure S2. Representative FCS curves of 594neg-SM (Lo phase marker) and 594neg-DOPC (Ld phase marker) in Lo-Ld phase-separated GUVs composed of SM/DOPC/Chol (1:1:1) in the absence (red) and presence of 5.4 nmol of AM3 (blue).
